# 12 tips for developing inter-professional education (IPE) in healthcare

**DOI:** 10.15694/mep.2019.000069.1

**Published:** 2019-03-26

**Authors:** Elaine Hill, Erin Morehead, Dawne Gurbutt, Joanne Keeling, Morris Gordon

**Affiliations:** 1 University of Central Lancashire (UCLan)

**Keywords:** Interprofessional, education, patient safety, healthcare, collaboration, curriculum, nursing, medicine, allied health

## Abstract

This article was migrated. The article was marked as recommended.

As healthcare increases in complexity there is growing awareness that interprofessional teamwork underpins safe and effective care delivery. However, in order to collaborate in interprofessional teams, health professionals must also train in them. Despite increasing interest in IPE amongst healthcare educators, and positive comments from students, barriers to its implementation remain. The authors of this article come from different healthcare professions and have overcome the challenges of developing IPE to devise several successful activities. This article outlines the educational benefits of IPE and provides guidance for surmounting obstacles to its implementation, supported by examples from our own experience.

## Introduction

IPE ‘occurs when two or more professions learn about, from and with each other to improve collaboration and the quality of care’ ( CAIPE, 2002) and has the potential to positively change healthcare cultures by influencing practitioners’ interactions ( WHO, 2010; Reeves *et al*, 2013). Healthcare staff work in multi-professional teams, where respect and collaborative working are essential for safe and effective patient care ( Aizer *et al.*, 2012; Gjessing *et al.*, 2014; El-Awaisi *et al.*, 2017; Ruebling *et al*., 2014; Trossman, 2014). This is increasingly important as healthcare services become more fragmented and the number of patients with complex healthcare needs rises ( Olson and Bialocerkowski, 2014; WHO, 2010; Little *et al.*, 2012). IPE is generally supported by professional healthcare bodies (CHMS, 2003; GMC, 2015; NMC, 2018; HCPC, 2015) and highly rated by students ( Lie *et al.,* 2013; Hammick *et al*, 2007). However, it is often neglected by educators, with lack of funding and management support, high student numbers, scheduling issues, lack of time and interest, lack of knowledge and skills, lack of confidence and structural barriers, being commonly cited reasons ( Kirsch and Ast, 2015; Anderson, 2016). Consequently IPE is frequently undertaken voluntarily or presumed to occur in the practice elements of courses, rather than integrated into programmes, according it reduced importance ( Rodger and Hoffman, 2010).

This article offers some practical tips for overcoming barriers and developing IPE activities.

## Tip 1: ‘Start networking’

Like-minded colleagues are vital, both for mutual support and developing appropriate IPE opportunities and materials. Although universities contain a wealth of knowledge and skills, they are frequently ‘siloed’, so staff and students rarely cross the professional or disciplinary barriers spontaneously ( Lloyd, 2016). We have overcome this by knocking on doors and telephoning or emailing colleagues we did not know, based on their job titles. Most people respond positively and if they cannot help, they often know someone who can. Staff meetings, internal/external conferences, away days or departmental emails have also resulted in exciting collaborations.

Ultimately, establishing an IPE interest group helped us to provide peer support, share expertise and encourage innovation and creativity. Research shows that educators generally require training and preparation to undertake IPE ( Hammick *et al.,* 2007; WHO, 2010) and relate to different student professionals ( Darlow *et al.,* 2017) as they may initially lack confidence with new teaching approaches and different learners. An IPE group can also help educators to overcome commonly-cited resistance from management for IPE initiatives ( Hammick *et al.,* 2007; WHO, 2010). Our organisation has now appointed two dedicated faculty IPE leads to support developments, alongside a university lead for inter-disciplinary education. In addition, one author is supporting various overseas colleagues to develop IPE activities and local networks.

Finally, we strive to ‘practice what we preach’ and deliver IPE in inter-professional teams. Each member contributes different qualities and knowledge, enabling us to better understand and appreciate one another’s perspectives, provide peer support and act as role models for learners ( Gurbutt and Keeling, 2018; Teodorczuk *et al*, 2016).

## Tip 2: ‘Pick a topic that different professionals engage in together in the “real world”’

For IPE to be appealing, meaningful and relevant to learners it must be authentic, so it is essential to assemble groups of professionals in relevant educational scenarios who will also work together practice ( Hammick *et al.,* 2007). It is also important to recognise and use learners’ different levels of prior knowledge and experience ( Kilminster *et al.,* 2004). We have successfully delivered IPE around cardiovascular disease for pharmacy and nursing students, by utilising the former’s understanding of medication and the latter’s knowledge of blood pressure monitoring, and involving the students as both teachers and learners in the session. We have also collaborated with outside organisations, actors and service users to deliver IPE targeting healthcare provision for homeless people and drug users - and management of different physical conditions - for students from a wide variety of disciplines ( Gurbutt and Milne, 2016; Gurbutt and Milne, 2018: Gurbutt and Milne, 2019). We find such activities generate novel, practical solutions to problems and promote the development of essential non-technical skills such as decision-making, problem solving, teamwork and communication, which are vital for patient safety ( Gordon, Darbyshire and Baker, 2012; Gordon *et al.,* 2019).

## Tip 3: ‘Focus on a topical or relevant issue’

Appropriate IPE topics may be of local and/or national importance e.g. safe drug administration. Whilst there are both international ( WHO, 2011) and national (e.g. DoH, 2007) drivers for this, specific local concerns may vary e.g. the design of prescription charts may be a potential source of error in one location and interruptions to administering staff in another. Cross-curricular topics may assume greater significance when taught inter-professionally ( Hammick *et al.,* 2007). For example, we found both undergraduate nurses and Operating Department Practitioners (ODPs) may fail to appreciate accountability and responsibility regarding medicines administration when taught in mono-professional groups, deeming knowing correct medication doses, or checking that a prescription is correct, as the sole responsibility of the prescriber. When placed into context through IPE with law students, learners more readily comprehend their responsibilities and the potential consequences of drug errors for themselves, as well as patients. Discussing these issues in IPE groups with their peers may also be more effective at dispelling incorrect beliefs than the same information provided by a healthcare educator!

IPE has also engendered collaboration between our university and NHS providers to address local issues e.g. co-creating an event to raise dementia awareness, which has since been shared with other care providers.

## Tip 4: ‘Look for ‘natural’ IPE topics in curricula’

IPE is effective for topics which are important, but often overlooked. For example, handover skills are required by all healthcare professionals but despite being mentioned in our curricula, no specific handover education was offered. This is a crucial oversight as poor handover is associated with healthcare errors and potential patient harm ( WHO, 2007; Keogh, 2013; DeKosky *et al.,* 2013; Benham-Hutchins and Effken, 2010). Recent systematic reviews of the handover education literature ( Gordon and Findley, 2011; Gordon *et al*, 2018) revealed a paucity of handover education in healthcare programmes and poor standards when it is undertaken. This combination of factors made it relatively easy to obtain funding and staff engagement to develop and pilot an IPE handover workshop for undergraduates from several healthcare professions. This resulted in a statistically significant increase in self-reported confidence, skills and knowledge in performing handover ( Hill, Gordon and Gurbutt, 2017).

## Tip 5: ‘Don’t try to include every profession’

In our experience learners will disengage from IPE if too many professions are included or groups are professionally unbalanced (e.g. 17 nurses, one medical student and one pharmacist) as activities or scenarios can become contrived and unrealistic. Students also report less satisfaction when undertaking IPE with professionals they would not normally collaborate with in practice ( Morehead, Lawrenson and Hill, 2018).

In real life inter-professional working rarely involves all professions, so educational situations must mirror this to be credible. We have found that a maximum of 4 or 5 professions is ideal, and it is essential that students adopt their own professional roles. Including service users and stakeholders as part of the group may also be beneficial ( Gurbutt and Milne, 2016). Over the course of a programme students may work in a number of inter-professional groups, either for different topics or through revisiting previous topics in a spiral curriculum. We find that this prevents IPE from becoming ‘formulaic’ and maintains interest.

## Tip 6: ‘Make sure it is IPE and not merely shared learning’

Although they are distinctly different the terms ‘shared learning’ and IPE are often used interchangeably in the published literature ( Olenick, Allen and Smego, 2010; McPherson, Headrick and Moss, 2001). Unlike IPE shared learning involves different professions learning together, but in the absence of collaboration ( Skinner, 2007; Goble, 2004) and may be used to reduce demands on resources or due to misunderstandings about what constitutes IPE ( Mazhindu, 2001). Whilst students perceive some benefits ( McComas, Doctor and Inglehart, 2019), and it may act as a catalyst for developing IPE, it does not result in them learning ‘from’ participant interactions ( Hammick *et al.,* 2007; Miller, Ross and Freeman, 1999; Gurbutt and Milne, 2018).

This concurs with socio-material theory which views individuals as inseparable from their social and material relationships, on which their learning and knowing are predicated ( McMurty, Rohse and Kilgour, 2016). Most healthcare errors arise from miscommunications within teams, rather than individuals’ incompetence or inattention ( Mazhindu, 2001). Learning from one another supports the development of trust and genuine teamwork, rather than ‘crew’ training ( Arrow and Henry, 2010), enabling members to function better collectively than individually.

To stay focussed, we also find it helpful to ‘benchmark’ planned IPE sessions against the CAIPE (2002) definition.

## Tip 7: ‘Consider the timing and the time needed’

Students must establish their own professional identities - through profession-specific learning - whilst simultaneously learning to collaborate (HCPC, 2015; Hammick *et al.,* 2007) (see Tip 10), so the timing of IPE is crucial. There are three issues to consider when scheduling IPE - level of professional development, curriculum and the academic calendar.

Commencing IPE prior to practice placements has long been recognised as most appropriate for undergraduates ( Castro, 1987), and favoured by them ( Lie *et al.,* 2013), as it prepares them to benefit from collaborative learning opportunities which arise in the clinical environment ( Joynes, 2018). Frequent sessions ( Bridges *et al*, 2011) in smaller groups ( Telford and Senior, 2017) generate greatest engagement.

The timing of topics in curricula may vary between professions, requiring IPE activities to accommodate students from different years of study. This is a not problematical provided that learners have clear ground rules and expectations (McPherson, Headrick and Moss, 2001). Programmes incorporating ‘spiral’ curricula, whereby the same topic is revisited at different levels in successive years (e.g. UCLan, 2018), offer considerable IPE opportunities as different professions can be included on each circuit of the spiral. For example, fundamental handover skills could be developed in year 1 with nursing and paramedic students then handover could be embedded within a multi-professional sepsis scenario in year 3 involving pharmacy, medicine, nursing, and ODP students.

Scheduling IPE over the academic year can be challenging due to large students numbers, working across programmes and staff specialities and needing specific types or sizes of rooms ( Hammick *et al*, 2007). Further, some learners e.g. UK nursing students are not bound by normal academic terms. Timetabling issues are frequently cited as reasons for avoiding IPE or failed attempts ( Pittenger, 2013) but we have found that it can be accommodated with some judicious lateral thinking and ingenuity.

Finally, one of the greatest barriers to IPE can be workload issues for staff, who frequently juggle varied pedagogical, institutional and professional body requirements in relation to teaching. Imposing IPE when they lack the necessary time for proper preparation and delivery may result in staff paying it ‘lip service’ ( Bridges *et al.,* 2011; Joynes, 2018). Dedicated staff, with specific skills and interests in IPE, can help address this problem.

## Tip 8: ‘Offer it as an “optional extra” to begin with’

Although IPE should ideally be integrated into curricula ( Teodorczuk *et al.,* 2016; Mazhindu, 2001; Pittenger, 2013; Stone, 2010; Ebert *et al.,* 2014) offering it as an extracurricular activity can be a useful starting point ( James *et al.,* 2017; Brooks *et al.,* 2017) and encourages course leaders and managers to include it in curricula. 85% of students who we surveyed stated they would attend an optional IPE event, even if scheduled outside of normal teaching hours ( Morehead, Lawrenson and Hill, 2018). We piloted an optional handover education workshop with undergraduate nurses, pharmacists, paramedics, operating department practitioners and doctors. Students were released from practice to attend and places were limited, which avoided timetabling issues and managing large student numbers. Staff used their research and scholarly activity time to participate and the workshop ran at the end of semester 2, when rooms were readily available. Students found it beneficial for both their practice, and to help them stand out in a competitive job market ( Hill, Gordon and Gurbutt, 2017). Further extracurricular IPE activities have since been offered.

University management and leadership are acutely sensitive to student feedback, which may be important in informing local - and potentially national - policy regarding the inclusion of IPE in educational programmes ( WHO, 2010; Hammick *et al*, 2007). Positive student evaluations of our handover education workshop helped to generate interest at a local NHS Trust and feedback on IPE around health issues for homeless people and drug users resulted in its inclusion in the newly validated MSc Occupational Health.

## Tip 9: ‘Enable students to discuss and evaluate with each other outside of the activities’

In our experience learning often occurs outside of prescribed IPE activities e.g. during breaks and lunch ( Morison *et al.,* 2003; Mu *et al.,* 2004) which can reinforce formal input and enhance positive attitudes towards other participants and professions ( Hammick *et al*., 2007). As informal social interactions are potentially as important as the actual IPE activities ( Nash and Hoy,1993; Reeves, 2000), allowing them sufficient time to develop in a meaningful way is essential. These observations are consistent with the situated learning model ( Lave and Wenger, 1991; Merrian, Cafarella and Baumgartner, 2007) which considers learning as inseparable from the context in which it occurs and generated through the social interactions of the learners ( Zakrajsek and Schuster, 2018). Learning may also continue through reflection once activities have concluded ( Palis and Quiros, 2014).

We find relationships between groups of students develop early in IPE activities and may continue beyond; interprofessional student support for programme validation and a local Health Mela are just two examples. Such collaborations between students can benefit academic programmes, student confidence and local communities ( Hoffman *et al*, 2008). This impact of IPE cannot be engineered, but is more likely to thrive when learners have space for connection.

## Tip 10: ‘Manage professional identities appropriately’

Professional identity is defined by membership of a specific professional group and the boundaries between this and other professional groups ( Burke, 2004; Best and Williams, 2018). It is individually constructed ( Lane, 2018; Best and Williams, 2018) and engenders self-esteem and belonging ( Tajfel and Turner, 1986) which subsequently determine behaviours and attitudes ( Siebert and Siebert, 2005). Identity formation may begin once a particular career is chosen ( Joseph *et al.,* 2017) and continues to develop during formal training ( Frenk *et al*, 2010). It changes little once established ( Best and Williams, 2018) but may be more malleable earlier in a career ( Ibarra, 1999).

Professional identity is challenged by new roles e.g. physician’s assistants which cross traditional professional boundaries and by staff from established professions whose roles more readily align with those outside of their profession ( Joynes, 2018). It seems logical that threats of change and uncertainty may reinforce existing professional boundaries and stereotypes as a form of self-protection.

Interprofessional teams must collaborate whilst simultaneously maintaining their discrete professional identities ( Hornby and Atkins, 2000; Joynes, 2018; Kvarnström, 2008). Teams challenge professional identities in three ways. Firstly, members who fear dilution of professional identities may resist co-operation ( Pate, Fischbacher and McKinnon, 2010). Secondly, diversity within interprofessional teams may be perceived as threatening ( Mitchell, Parker and Giles, 2011; Holmesland *et al*, 2010). Thirdly, professional hierarchies may form barriers to interprofessional working (Best and Williams, 2008). The solution is enabling staff to develop a ‘dual identity’, as members of both specific healthcare professions and an interprofessional team ( Khalili *et al.,* 2013; DiVall *et al.,* 2014; Best and Williams, 2018), alongside overcoming cultural barriers to collaboration by developing understanding of other professionals’ work ( Mitchell, Parker and Giles, 2011; Holmesland *et al*, 2010).

It logically follows that healthcare educators must nurture dual identities in their students by providing both mono-professional and interprofessional education throughout their programmes. This may enable learners to understand their professional boundaries, and their contributions to an interprofessional team, without these boundaries developing into barriers, as they do not feel their territories are threatened. This may also ease the acceptance of those in new professions and discredit negative professional stereotypes ( Hammick *et al.,* 2007).

An example from our experience involves pharmacy and physiotherapy students participating in IPE with the shared goal of reducing a patient’s pain, but different methods for achieving this. The realisation that they complemented one another’s’ skills and knowledge, rather than threatening one another’s space, allowed them to address the problem more effectively through collaboration than individually.

## Tip 11: ‘Consider resources (e.g. staff, facilities, equipment)’

Using less resources - or supplementing resource-intensive activities with simpler options ( Teodorczuk *et al.,* 2016) - may make IPE more attractive as costs and complexities are reduced; this may be especially important initially. Our IPE handover workshop used a standard classroom, flipcharts/pens, PowerPoint/film, three lecturers plus some printed resources with costs estimated at £430 for 40 students. Conversely, a high-tech simulation laboratory and disposable equipment costs considerably more, and fewer students can be included per session. All types of IPE have their place but keeping things simple and low cost, in return for positive outcomes, may help persuade budget holders and sceptics to support IPE developments ( Hammick *et al.,* 2007; WHO, 2010). For our students the authenticity of the IPE scenario was more important than the level of fidelity used ( Morehead, Lawrenson and Hill, 2018).

## Tip 12: ‘Seize any opportunities for support’

Relevant interprofessional educational materials on a topic may already exist, which can be used or adapted. For example, WHO (2011) has designed teaching materials for infection control/prevention and improving medication safety in several languages whilst the NHS III (2010) has produced numerous videos/resources for teaching handover/escalation using SBAR. In both cases materials are free and readily available.

Whilst not always essential, published research indicates that more influential IPE developments are supported by specific funding ( Hammick *et al.,* 2007). We developed and piloted our IPE handover workshop using a £1000 internal award and also used it as small-scale research to generate evidence for supporting ongoing development of IPE within our institution. This can now benefit the wider IPE community as we share our experiences and workshop materials with others.

## Conclusions

In conclusion, IPE is beneficial for learners and achievable by educators. We hope that these tips will prove helpful to educators seeking to undertake effective IPE activities with students from a range of healthcare professions.

## Take Home Messages

• IPE is a valuable tool for developing collaborative healthcare practitioners and enabling them to deliver safe and effective patient care.
• Educators can overcome barriers to undertaking IPE, and create successful IPE activities, through co-operation and ‘thinking outside of the box’.

## Notes On Contributors

Elaine Hill is a Senior Lecturer with strong research and teaching interests centred on interprofessional education and collaboration, and their role in patient safety, both locally and internationally. She is a Registered Adult Nurse and Operating Department Practitioner with a background in anaesthetic and recovery practice. https://orcid.org/0000-0003-4984-9446

Erin Morehead is Principal Lecturer in Academic Development at UCLan and the Academic Development Lead for Life Sciences and Simulated Learning. She has a keen interest in interprofessional collaboration and education. She is a Chartered Physiotherapist with a background in sport. https://orcid.org/0000-0002-4096-0220

Dawne Gurbutt is Professor of Collaborative Learning and Teaching at UCLan. Committed to collaboration and building communities, she works across traditional boundaries developing connections and educational opportunities. She is a Registered Nurse with a background in community nursing.

Joanne Keeling is the Pre-Registration Nursing Education Manager and Faculty of Health and Wellbeing lead for inter-professional education. She is a Registered Mental Health Nurse and a keen advocate of mental health and wellbeing promotion and collaborative approaches to learning and teaching. https://orcid.org/0000-0002-0151-7234

Professor Morris Gordon is a paediatrician and holds a chair in evidence synthesis and systematic review. He is the chair of the Best Evidence Medical Education Executive and has completed several reviews on topics in education. https://orcid.org/0000-0002-1216-5158

## Declarations

The author has declared that there are no conflicts of interest.

## Ethics Statement

No ethics approval required as this paper only involves reviewing literature and our own experiences as educators.

## External Funding

This article has not had any External Funding
