# Supplementary material for: 12 tips for developing inter-professional education (IPE) in healthcare
Source: MedEdPublish (2016). 2019 Mar 26;8:69. [Version 1] doi: 10.15694/mep.2019.000069.1 (PMC10911769; doi:10.15694/mep.2019.000069.1)
Supplement: Peer review history

# Reviewer report: Reviewer response for version 1

**DOI:** 10.21956/mep.19801.r29790

**Published:** 2019-07-30

**Authors:** Jayne Garner

**Affiliations:** 1 university of liverpool

This review has been migrated. The reviewer awarded 4 stars out of 5 Useful and practical, these 12 tips on IPE are clearly presented and supported by a good range of evidence and professional insight. In particular, the points applying IPE to 'real world' situations and managing professional identities were helpful for planning sessions. Thank you for sharing this paper.

Reviewer Expertise:

NA

No decision status is available

# Reviewer report: Reviewer response for version 1

**DOI:** 10.21956/mep.19801.r29787

**Published:** 2019-07-26

**Authors:** Shelley Fielden

**Affiliations:** 1 Leeds Institute of Medical Education

This review has been migrated. The reviewer awarded 4 stars out of 5 A well written article that will be of interest to curriculum developers looking to develop interprofessional learning opportunities within their own School. I was pleased to see the author team recognise the value of modelling interprofessional practice within their own team when discussing curriculum development and delivery, this has been a key benefit to the approach in my own organisation. Within universities it is often difficult to avoid developing IPE that includes all programmes given increasing regulatory requirement of regulators in the UK to include IPE within their degree programmes. I agree with the authors that this makes ensuring clinical realism and relevance problematic. One issue that was not discussed but may be helpful to others is to recognise the different levels of resource (particularly around funding) across different programmes and Schools as for curriculum developers to be creative in sharing resource in order to collaborate on IPE.

Reviewer Expertise:

NA

No decision status is available

# Reviewer report: Reviewer response for version 1

**DOI:** 10.21956/mep.19801.r29789

**Published:** 2019-04-29

**Authors:** Saee Deshpande

**Affiliations:** 1 Maharashtra University of Health Sciences, Nashik- VSPM Dental College Nagpur

This review has been migrated. The reviewer awarded 4 stars out of 5 Tips are quite comprehensive and useful for those who wish to implement IPS in their set-up.In my experience the factors of 'Motivation' and 'Sense of belonging in the Team'are equally important for the success of such initiatives and thus tools to increase the same amongst the team members will go a long way.Very well written indeed.

Reviewer Expertise:

NA

No decision status is available

# Reviewer report: Reviewer response for version 1

**DOI:** 10.21956/mep.19801.r29788

**Published:** 2019-03-31

**Authors:** Balakrishnan Nair

**Affiliations:** 1 Centre for Medical Professional Development and University of Newcastle

This review has been migrated. The reviewer awarded 4 stars out of 5 IPL is a very important component of health professional education and as the authors state, well accepted by learners and teachers. Unfortunately , it is still difficult to implement because of lack of co-operation among disciplines , time tabling problems and poor funding. Any body trying to establish IPL will find these tips useful and anybody providing IPL will agree with the evidence presented here. It is well written and referenced.

Reviewer Expertise:

NA

No decision status is available

# Reviewer report: Reviewer response for version 1

**DOI:** 10.21956/mep.19801.r29786

**Published:** 2019-03-26

**Authors:** Megan Anakin

**Affiliations:** 1 University of Otago

This review has been migrated. The reviewer awarded 4 stars out of 5 Thank you for writing this information-packed article. I appreciate that the 12 tips are well-supported by references to a broad range of the current literature and illustrated with your experiences. Each tip contains several ideas to prompt reflection. I would recommend this article to educators new to IPE who are interested in developing an IPE activity. I would also recommend this article to experienced IPE educators to help them consider the strengths and areas for development in their IPE programmes.

Reviewer Expertise:

NA

No decision status is available
